# Evaluation of the Association between Gestational Diabetes Mellitus at First Pregnancy and Cancer within 10 Years Postpartum Using National Health Insurance Data in South Korea

**DOI:** 10.3390/ijerph15122646

**Published:** 2018-11-26

**Authors:** Kyu-Tae Han, Geum Joon Cho, Eui Hyeok Kim

**Affiliations:** 1 Research and Analysis Team, National Health Insurance Service Ilsan Hospital, Goyang 10444, Korea; kthan.phd@gmail.com; 2 Department of Obstetrics and Gynecology, Korea University College of Medicine, Seoul 08308, Korea; md_cho@hanmail.net; 3 Department of Obstetrics and Gynecology, National Health Insurance Service Ilsan Hospital, Goyang 10444, Korea

**Keywords:** gestational diabetes mellitus, diabetes mellitus, cancer, thyroid cancer

## Abstract

This study aims to evaluate the association between gestational diabetes mellitus (GDM) at first pregnancy and the incidence of cancer within 10 years postpartum. We used customized health information data from the National Health Insurance Corporation (NHIC). This retrospective cohort study included data from women who were not previously diagnosed with diabetes or any kind of malignancy in the National Health Screening Examination through the NHIC during 2002–2003, and only women who had their first delivery between 2004 and 2005 was included. Follow-up cancer diagnosis was carried out up until 2015. Among the 102,900 primiparous women, 4970 (4.83%) were diagnosed with GDM. During 10 year total follow-up period, 6569 (6.38%) cases of primary cancer were identified. The incidence of cancer was higher in women with GDM and the most common type of cancer was thyroid cancer, followed by breast cancer. On the basis of survival analysis, we adopted the Cox proportional hazards model and found that GDM was positively associated with cancer, particularly in thyroid cancer (HR: 1.27, 95% CI: 1.054–1.532, *p* = 0.012). However, the incidence of other malignancies (including ovarian and breast cancers) were not significantly associated with GDM, though they did show positive trends. Our findings suggest that GDM is associated with the incidence of cancer, particular thyroid cancer.

## 1. Introduction

Gestational diabetes mellitus (GDM) is defined as a carbohydrate intolerance of variable severity with onset or first recognition occurring during pregnancy [1,2]. The prevalence of GDM is about 6–7% in the United States and 5.6% in South Korea, and the global prevalence of GDM is increasing [1,3,4,5]. Pregnancy is normally accompanied by progressive insulin resistance due to a combination of increased maternal adiposity and the insulin-desensitizing effects of diabetogenic placental hormones, such as the human growth hormone, corticotropin-releasing hormones, placental lactogen, and progesterone [6]. Although most pregnant women are able to compensate for this condition through increased insulin secretion, women with GDM become hyperglycemic, which may cause various chronic metabolic abnormalities [7].

After delivery, the hyperglycemic effects of placental hormones dissipate rapidly and most women with GDM revert back to their pre-pregnancy glycemic status almost immediately; however, these women are more likely to have insulin resistance and hyperinsulinemia, predisposing them to risk factors for the development of type 2 diabetes mellitus (DM) later in life [8,9,10]. It was reported that women with a history of GDM have a 7-fold higher risk of developing DM compared to women with normal glucose tolerance during pregnancy [10]. In addition, some studies reported an association between type 2 diabetes and a higher risk of certain types of cancers [11,12]. The mechanisms are yet to be investigated, but insulin resistance with secondary hyperinsulinemia is the most frequently proposed hypothesis because insulin might have a mitogenic effect by binding to the insulin-like growth factor-I receptor [12,13,14]. Further, hyperglycemia itself might promote carcinogenesis by increasing oxidative stress [15,16,17]. According to previous studies, there are several positive associations between overt DM and malignancies of the pancreas, colon, and liver [18,19,20,21,22]. Among women, strong evidence links endometrial cancer with DM, but associations of breast and ovarian cancers with DM have not been fully established [23,24,25]. Data regarding the cancer risk associated with GDM are rare and limited to cancers of the breast and pancreas [26,27].

Although the incidence of GDM has gradually increased and related issues could be emerging in South Korea in the near future, there exists a lack of study on the relationship between GDM and other diseases in South Korea. Thus, the present study aims to evaluate the association between GDM at first pregnancy and the incidence of primary cancer within 10 years postpartum by using insurance claim data in South Korea, and to determine the short-term risk of future cancers in young women with GDM compared to those without GDM.

## 2. Methods

### 2.1. Study Design and Population

In South Korea, about 97% of the population is obligated to enroll in the National Health Insurance Service (NHIS) and the other 3% are covered by medical care to protect them from the financial burden of excessive medical expenditure. Therefore, the NHIS database contains information on all medical claims for approximately 50 million Koreans, including NHIS and medical care beneficiaries, and nearly all information about the extent of a disease can be obtained from this centralized database. In addition, as part of the NHIS healthcare program, beneficiaries who satisfy a specific criteria (self-employed beneficiaries are insured, beneficiaries of NHI employee insurance, and dependent families [more than 40 years old] with beneficiaries of NHI employee insurance) are invited to participate in a biannual National Health Screening Examination (NHSE), and their screening results are recorded in the NHIS database.

This study used customized health information from the National Health Insurance Cooperation that could be modified as requested for the purpose of policy and academic research (https://nhiss.nhis.or.kr). Using this database during 2002–2015, we identified all women in South Korea who had their first delivery between 1 January 2004 and 31 December 2005 (Figure S1 in Supplementary Materials). To facilitate the evaluation of the pre-pregnancy characteristics, women were only included in the analysis if they underwent an NHSE at least 2 years before their first delivery; the NHSE consists of a health interview and a health examination. The health interview included questions regarding patients’ demographic, socioeconomic and lifestyle characteristics. To investigate the association between GDM and cancer in women after their first delivery, we excluded those with pre-existing type 1 DM and diagnosed type 2 DM before delivery based on the International Classification of Disease Tenth Revision codes (ICD)-10 (O24.0, O24.1, O24.3 and E11). We did this before their first delivery in order to facilitate the evaluation of the pre-pregnancy characteristics. Additionally, we excluded women who were diagnosed as having cancer during 2002–2003, as they were assumed to be newly diagnosed as having cancer. Finally, 102,900 women who experienced their first delivery were included in this study. This study was approved by the Ethics Committee of the National Health Insurance Service, Ilsan Hospital (approval number: NHIMC 2017-01-012).

### 2.2. Primary Outcomes and Pre-Pregnancy Factors

One primary outcome was whether women were diagnosed as having cancer after their first delivery. The diagnosis of cancer was defined according to ICD-10 codes (Supplementary Figure S2); in this study, we evaluated overall cancer and each type of cancer. If women visited a medical institution because of cancer after delivery we defined them as patients with cancer.

The other primary outcome of interest was a diagnosis of GDM after first delivery. Women with GDM during their first pregnancy were identified by a principal or secondary diagnosis of GDM based on ICD-10 codes (O24.4 and O24.9).

We also included the following pre-pregnancy factors from the NHSE data to adjust for the differences between primiparous women: Age, body mass index (BMI), smoking status and fasting blood glucose (FBG) level. To evaluate the difference in association between thresholds, we considered age and BMI as categorical variables [28,29]. Age was divided into quartiles: Q1, less than 24 years; Q2, 25–29 years; Q3, 30–34 years; and Q4, more than 35 years. BMI was calculated using height and weight measurements and was divided into four categories: Low weight, normal weight, overweight and obesity, according to cutoff values of 23 kg/m^2^ and 25 kg/m^2^ for Korean adults, which were proposed by the Korean Society in its study on obesity [30]. Smoking status was categorized as current, ex-smoker, and non-smoker. If a woman knows that she has a high FBG level, she may make an effort to manage her clinical status and adopt healthy behaviors; this may make a difference in the diagnosis of cancer due to GDM. Thus, we included FBG before pregnancy as an independent variable in this study.

### 2.3. Statistical Analysis

We first examined the general characteristics of the study population, where continuous and categorical variables are expressed as a mean and frequency, respectively. Thereafter, we performed the chi-square test to examine the difference in the distribution of general characteristics and types of cancer according to the diagnosis of GDM. We also performed an analysis of variance (ANOVA) to identify the mean and standard deviation of continuous variables, such as age, by the GDM separately. Next, the postpartum 10-year cumulative incidence of cancer was estimated using the Kaplan-Meier method and compared using the product-limit method with the log-rank test according to GDM. We also created a log-log plot to check the proportional hazards assumption of the Cox model before survival analysis using the Cox proportional hazard model. Finally, we performed survival analysis using the Cox proportional hazard model to estimate the adjusted hazard ratios (HRs) and a 95% confidence interval (CIs) for the diagnosis of cancer over 10 years postpartum. All statistical analyses were performed using SAS, version 9.2 (SAS Institute, Inc., Cary, NC, USA).

## 3. Results

**Figure 1:**
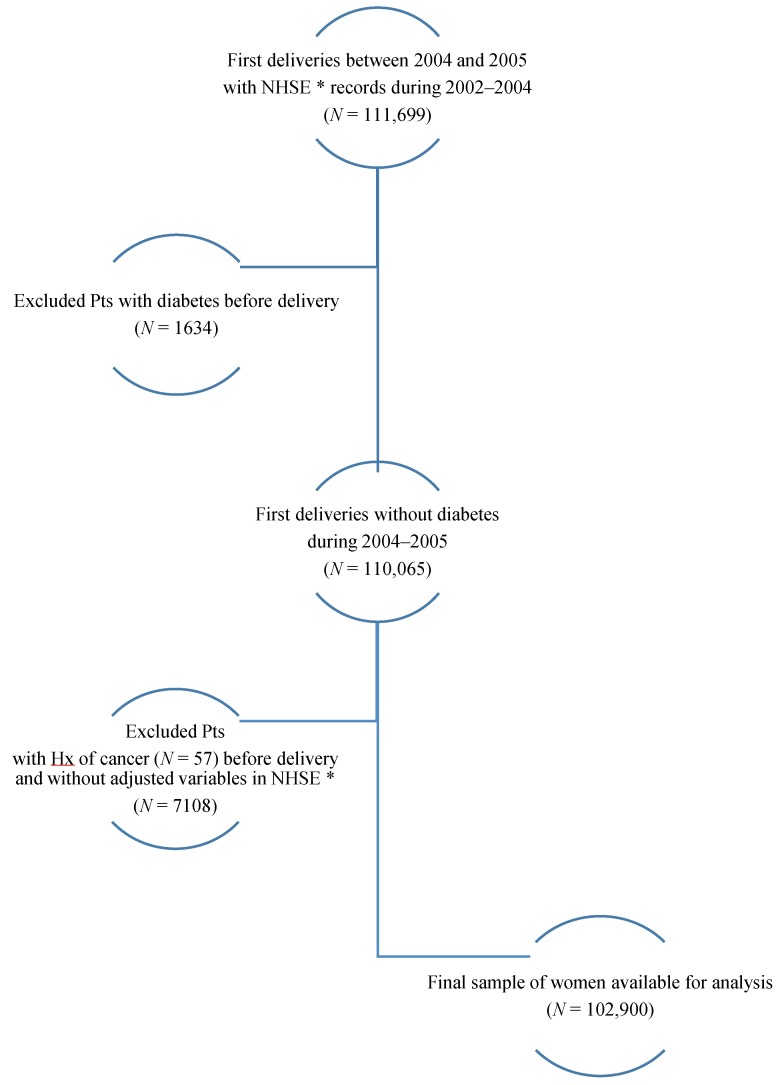
The study population in this study. * NHSE; National Health Screening Examination.

In South Korea, 111,699 women had their first delivery between 2004 and 2005 according to NHSE records from 2002–2004. We excluded 1634 women who were previously diagnosed as having diabetes, 57 with previous cancer and 7108 with missing values for the independent variables in the NHSE (i.e., age, BMI, smoking status, and FBG level). Finally, 102,900 women were included in this study (Figure 1). GDM occurred in 4970 (4.83%) of the study participants.

Table 1 shows the general patient characteristics and results of the chi-square test and ANOVA based on the diagnosis of GDM. Overall, 5617 (5.5%) primiparous women were diagnosed as having DM within 10 years postpartum. A diagnosis of GDM was more common in older women than in younger women and the proportion of women older than 35 years was significantly high compared to the rest of the population. In addition, more women with a higher BMI and FBG level were diagnosed as having GDM than those with a lower BMI and FBG level. Women with GDM were more associated with a diagnosis of DM after first delivery than those without GDM.

Figure S1 and S2 in Supplementary Materials show the Kaplan-Meier curves for the cumulative incidence of overall cancer and for each type of cancer by GDM. During the follow-up, the cumulative incidences of cancer were higher in women with GDM than in those without GDM (*p* < 0.0001). On the basis of the results by each type of cancer, thyroid cancer, breast cancer and hepatoma were also associated with GDM.

Table 2 shows the incidence of cancer overall and by each type of cancer, according to the diagnosis of GDM, showing the results of survival analysis using Cox proportional hazard models to estimate the adjusted HRs for cancer with respect to GDM. On the basis of the results, diagnosis of overall cancer was more frequent in patients with GDM than in those without GDM (2.64% versus 1.86%, *p* < 0.001). GDM was positively associated with an increased diagnosis of overall cancer (HR = 1.31, 95% CI: 1.19–1.45, *p* < 0.001). Regarding the results by each type of cancer, thyroid cancer was highly associated with GDM (HR = 1.27, 95% CI: 1.05–1.53, *p* = 0.0120). The other types of cancer were also positively associated with GDM, although the effect estimates were not statistically significant.

## 4. Discussion

It is well known that women with GDM are more likely to have metabolic syndrome, an atherogenic lipid profile and early vascular dysfunction at ≥3 months postpartum compared to women without previous GDM [30,31] and type 2 diabetes [9,10]. Herein, we also found that GDM was also associated with an increased risk of thyroid cancer and overall cancer. GDM was associated with cancer diagnosis, particularly thyroid cancer. Considering that cancer develops more often with increasing age, women with GDM could have been more likely to be diagnosed as having other malignancies if the follow-up period was longer than 10 years.

Several studies have also assessed different measures of insulin resistance or diabetes in pregnancy and their association with cancer incidence, and the authors reported mixed findings [32,33,34]. Indeed, there is some biological evidence to support an association between diabetes, insulin resistance, and thyroid cancer. For instance, increased FBG levels have been linked to a higher risk of thyroid cancer [35], and preclinical studies have shown direct carcinogenic effects of hyperinsulinemia on thyroid cancer cells [35,36]. One study of women with even mild GDM (i.e., a normal FBG level according to a glucose tolerance test) reported that approximately one third of them developed metabolic syndrome within 5–10 years of delivery [37].

As previously mentioned, the association between cancer and diabetes has been investigated extensively and most but not all studies found that diabetes is associated with an increased risk of several types of cancer. However, there could be alternative explanations for this relationship. For instance, the coexistence of obesity and a sedentary lifestyle could induce hyperinsulinemia, or diabetes itself might develop because of cancer. However, in terms of the relationship with GDM, this explanation is not applicable. Although diabetes risk is high in women with GDM and most women were not diabetic at that time, even glucose intolerance during pregnancy was found to be associated with an increased risk of malignant neoplasm. This may be related to the mechanisms of cancer development.

The result of the present study (that GDM is associated with thyroid cancer) is in agreement with that of a previous study [38]. Bejaimal et al. showed that a regular follow-up of women with GDM after childbirth can facilitate early diagnosis of various cancers including thyroid cancer. The early diagnosis of cancer will expedite the treatment of cancer in the future, increasing the probability of cure without disease progression, possibly providing an opportunity to reduce the national medical expenditure as a whole and on an individual basis, especially considering delays until the first delivery of a child and the increased prevalence of GDM in South Korea.

The strength of the present study lies in its population-based cohort, which included more than 100,000 women and had almost no patients lost in the follow-up process. This large population-based cohort suggests that our findings may be widely generalized. Moreover, to minimize and remove the effect of parity and previously diagnosed GDM or DM, we enrolled only women who delivered their first child without DM.

A few limitations should be considered when interpreting the present study’s findings. The diagnoses of GDM and cancer were based on insurance claims data from the KNHI claims database, which was designed for cost claim issues and not research purposes. Therefore, the main limitation is the validity of the diagnoses in this database, although the overall prevalence of the cancers in South Korea was similar to that in a previous study [39]. Next, the number of ovary cancers was higher than the occurrence of breast cancers in this study, although breast cancer occurs more frequently than ovary cancer in South Korea. This was because this study included relatively younger individuals. The prevalence of ovary cancer was greater than breast cancer among 15–24-year-old women according to Korean cancer registry data. In addition, the subjects in this study are limited to only people who participated in NHSE. Given that the participation rate in the NHSE was not substantially high and subjects of this study had different characteristics, there are some limitations that can be generalized to South Korea on the whole. Further, we were unable to consider variables for cancer staging as there was no information on staging in the database. Thus, we could not examine severity in detail using a cancer staging system, such as TNM (tumor, nodes and metastasis) staging or SEER (surveillance, epidemiology and end results) summary staging. Finally, we made an effort to reduce the impact of both high FBG and DM before first delivery, excluded the patients with DM at the study baseline, and considered FBG measured before delivery as an independent variable in this study.

## 5. Conclusions

In conclusion, our study’s results indicate that even after a relatively short-term follow-up period in postpartum women, GDM, although a transient symptom for metabolic dysfunction in pregnancy and glucose intolerance that resolves after delivery in most cases, was positively associated with an increased diagnosis of overall cancer and thyroid cancer. This information could be used to modify the risk factors of future malignancy. Cancer prevention and early detection by appropriate screening methods in patients with GDM are crucial components of clinical management and the evaluation of women. These results may also serve to further enhance follow-up and preventive strategies for cancer in GDM as well as in type 2 diabetes. Future studies to determine which cancers are associated with GDM in the long-term are required.

## Figures and Tables

**Table 1:** General Characteristics of study population by gestational diabetes mellitus (GDM).

Variables	GDM		*p*-Value
	Yes (*n* = 4970)	No (*n* = 97,930)	
Age (year)	28.25 (±3.28)	27.28 (±3.02)	<0.0001
AMA	3.98% (198)	1.64% (1608)	<0.0001
Diabetes after delivery	9.46% (470)	5.26% (5147)	<0.0001
BMI (Kg/M^2^)	20.98 (±2.74)	20.77 (±6.74)	<0.0001
Smoking	1.85% (92)	2.01% (1970)	0.0406
Fasting glucose	85.99 (±20.97)	85.09 (±17.44)	0.0032

Data are presented as *n* (%) or mean ±SD, *p*-value for the results of chi-square test or ANOVA; GDM: gestational diabetes mellitus; AMA: advanced maternal age (≥35 years old at delivery); BMI: body mass index.

**Table 2:** The incidence of cancer by GDM (gestational diabetes mellitus) and the results of survival analysis for cancer by GDM.

Variables	GDM ^†^		Unadjusted			Adjusted ^††^		
	Yes	No	HR	95% CI	*p*-Value	HR	95% CI	*p*-Value
Thyroid Cancer	131 (2.64%)	1822 (1.86%)	1.34	1.114–1.617	0.002	1.27	1.054–1.532	0.012 *
Gynecologic cancerother than ovary cancer	29 (0.58%)	388 (0.40%)	1.29	0.830–2.005	0.258	1.32	0.846–2.050	0.223
Ovary cancer	81 (1.63%)	1067 (1.09%)	1.28	0.938–1.738	0.12	1.23	0.901–1.673	0.193
Blood cancer	3 (0.06%)	30 (0.03%)	1.65	0.391–6.999	0.494	1.52	0.357–6.445	0.573
Gastric cancer	16 (0.32%)	234 (0.24%)	1.52	0.915–2.530	0.105	1.41	0.845–2.345	0.189
Hepatoma	21 (0.42%)	296 (0.30%)	1.57	0.986–2.509	0.057	1.5	0.939–2.397	0.09
Breast cancer	43 (0.87%)	646 (0.66%)	1.33	0.962–1.825	0.085	1.15	0.831–1.581	0.405
Colon cancer	20 (0.40%)	245 (0.25%)	1.45	0.874–2.411	0.1501	1.33	0.799–2.213	0.273
Total cancer	437 (8.79%)	6132 (6.26%)	1.39	1.261–1.531	<0.0001	1.31	1.192–1.449	<0.0001 *

^†^ Data are presented as *n* (%); ^††^ Adjusted for maternal age, smoking, BMI before pregnancy and FBG. * Statistically significant, HR; hazard ratio.

## Supplementary Materials

The following are available online at http://www.mdpi.com/1660-4601/15/12/2646/s1, Figure S1: The EDI code for participants during 2004–2005, Figure S2: ICD-10 code for Cancer.
