# Supplementary material for: Evaluation of the Association between Gestational Diabetes Mellitus at First Pregnancy and Cancer within 10 Years Postpartum Using National Health Insurance Data in South Korea
Source: Int J Environ Res Public Health. 2018 Nov 26;15(12):2646. doi: 10.3390/ijerph15122646 (PMC6313396; doi:10.3390/ijerph15122646)
Supplement: Supplementary file 1 [file ijerph-15-02646-s001.pdf]

Evaluation of the Association between Gestational Diabetes Mellitus at First Pregnancy and Cancer within 10 Years Postpartum Using National Health Insurance Data in South Korea

Kyu-Tae Han [1], Geum Joon Cho [2] and Eui Hyeok Kim [3,*]

Supplementary

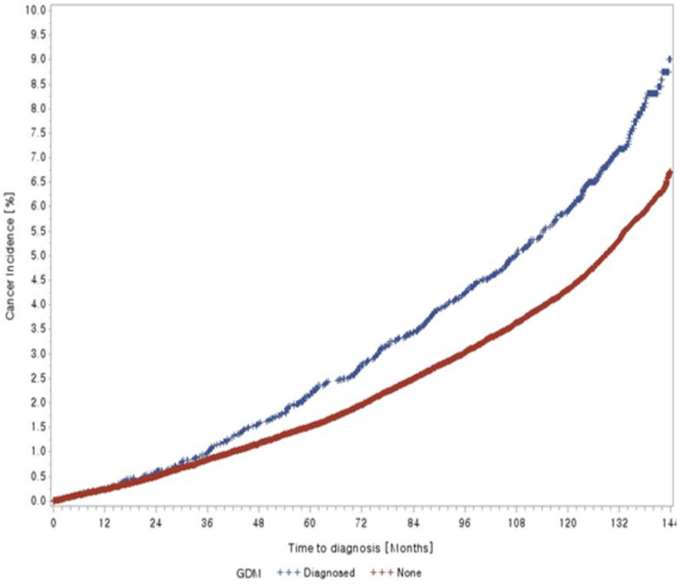

Figure S1. Kaplan-Meier Curves for incidence of total cancer by GDM. † p-value Log-rank test < 0.0001.

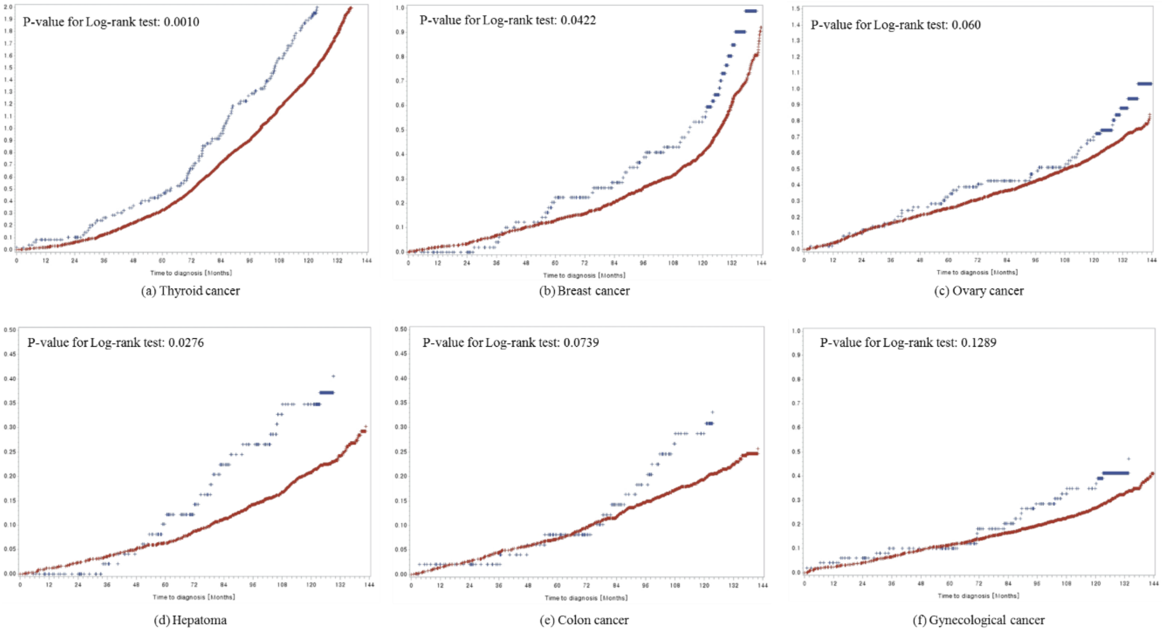

Figure S2. Kaplan-Meier Curves for each cancer by GDM.
